# Phenotypically-defined stages of leukemia arrest predict main driver mutations subgroups, and outcome in acute myeloid leukemia

**DOI:** 10.1038/s41408-022-00712-7

**Published:** 2022-08-16

**Authors:** François Vergez, Laetitia Largeaud, Sarah Bertoli, Marie-Laure Nicolau, Jean-Baptiste Rieu, Inès Vergnolle, Estelle Saland, Audrey Sarry, Suzanne Tavitian, Françoise Huguet, Muriel Picard, Jean-Philippe Vial, Nicolas Lechevalier, Audrey Bidet, Pierre-Yves Dumas, Arnaud Pigneux, Isabelle Luquet, Véronique Mansat-De Mas, Eric Delabesse, Martin Carroll, Gwenn Danet-Desnoyers, Jean-Emmanuel Sarry, Christian Récher

**Affiliations:** 1 Laboratoire d’Hématologie, Centre Hospitalier Universitaire de Toulouse, Institut Universitaire du Cancer de Toulouse Oncopole, Toulouse, France; 2 Université Toulouse III Paul Sabatier, Toulouse, France; 3 Cancer Research Center of Toulouse, UMR1037 INSERM, ERL5294 CNRS, Toulouse, France; 4 Stem Cell and Xenograft Core, University of Pennsylvania, Perelman School of Medicine, Philadelphia, PA USA; 5 Service d’Hématologie, Centre Hospitalier Universitaire de Toulouse, Institut Universitaire du Cancer de Toulouse Oncopole, Toulouse, France; 6 Laboratoire d’Hématologie, Centre Hospitalier Universitaire de Bordeaux, Pessac, France; 7 Service d’Hématologie Clinique et de Thérapie Cellulaire, Centre Hospitalier Universitaire de Bordeaux, Pessac, France; 8 Centre Hospitalier Universitaire de Toulouse, Institut Universitaire du Cancer de Toulouse Oncopole, Toulouse, France

**Keywords:** Acute myeloid leukaemia, Myelopoiesis

## Abstract

Classifications of acute myeloid leukemia (AML) patients rely on morphologic, cytogenetic, and molecular features. Here we have established a novel flow cytometry-based immunophenotypic stratification showing that AML blasts are blocked at specific stages of differentiation where features of normal myelopoiesis are preserved. Six stages of leukemia differentiation-arrest categories based on CD34, CD117, CD13, CD33, MPO, and HLA-DR expression were identified in two independent cohorts of 2087 and 1209 AML patients. Hematopoietic stem cell/multipotent progenitor-like AMLs display low proliferation rate, inv(3) or *RUNX1* mutations, and high leukemic stem cell frequency as well as poor outcome, whereas granulocyte–monocyte progenitor-like AMLs have *CEBPA* mutations, *RUNX1-RUNX1T1* or *CBFB-MYH11* translocations, lower leukemic stem cell frequency, higher chemosensitivity, and better outcome. *NPM1* mutations correlate with most mature stages of leukemia arrest together with *TET2* or *IDH* mutations in granulocyte progenitors-like AML or with *DNMT3A* mutations in monocyte progenitors-like AML. Overall, we demonstrate that AML is arrested at specific stages of myeloid differentiation (SLA classification) that significantly correlate with AML genetic lesions, clinical presentation, stem cell properties, chemosensitivity, response to therapy, and outcome.

## Introduction

Normal blood cell maturation is organized according to a functional hierarchy at the top of which are multipotent hematopoietic stem cells (HSC). Immunophenotypically, human HSCs are enriched in a population of Lin^−^, CD34^+^, CD38^−^, CD90^+^, and CD45RA^−^ cells [1–4]. Upon differentiation, HSC gives rise to multipotent progenitors (MPP), which retain the ability to produce all blood lineages but have lost their self-renewal capacity [4]. The classical model of hematopoiesis [5, 6] postulates that MPPs are then orientated toward either the lymphoid or myeloid lineage, developing into common myeloid progenitors (CMP) or lymphoid-primed multipotent progenitors (LMPP) which can still produce defined myeloid cell types. Along the myeloid pathway, CMP can differentiate into either granulocyte–monocyte progenitors (GMP) or megakaryocyte–erythroid progenitors (MEP). GMPs finally differentiate into granulocyte progenitors (GP) or monocyte progenitors (MP).

Acute myeloid leukemia (AML), the neoplastic counterpart of early hematopoiesis, is caused by the excessive proliferation of transformed hematopoietic progenitors which show great heterogeneity at the morphological, immunophenotypic, cytogenetic, and molecular levels [7]. Recent studies have shown that leukemic clones might develop from pre-leukemic hematopoietic cells carrying mutations in genes such as *DNMT3A*, *TET2*, or *ASXL1*, which then accumulate a series of secondary mutations some of which will block differentiation while others induce uncontrolled proliferation [8, 9]. Importantly, AML is initiated and maintained by rare and immunophenotypically diverse leukemic stem cells (LSC), phenocopying the hierarchical organization of normal hematopoiesis [10–12].

Despite a better characterization of AML and higher efficacy of new AML therapies, biomarkers for response prediction are currently lacking, in part because genomic aberrations have shown only limited predictive value [13]. More accurate response predictions, which go beyond genomics, are needed for some of the newest approaches to AML treatment.

The proteome and the surfaceome have been considered a promising and complementary fields to the genome for elucidating cancer biology and identifying diagnostic and predictive biomarkers [14, 15]. However, there are still very few clinically relevant biomarkers and disease subtypes that have been identified by these approaches [16–18].

Here, we did a phenotypic, cytogenetic, and molecular correlative analysis of a very large cohort of AML patients to capture the AML surfaceome that appears to be caused by the stage of leukemia arrest (SLA) and by specific genomic features. We hypothesized that our approach may add to current AML classifications by identifying relevant phenotypic AML subtypes with specific clinical and molecular features as well as information on outcomes after standard treatment. Overall, we anticipated that phenotypic classification associated with the morphological analysis, available within 24 h on the day of diagnosis, could be very useful to begin planning therapeutic strategy in daily practice.

## Patients and methods

### Patients and samples

Between January 1, 2000 and December 31, 2019, 2448 AML patients (>15 years) were included in the AML database of Toulouse University Hospital (TUH) and 1700 AML patients in the AML database of Bordeaux University Hospital (BUH). Immunophenotyping at diagnosis was available for 2087 TUH patients (median age: 63 years) of which 1266 received intensive chemotherapy [19, 20] (Table 1) and 1209 BUH patients (Table S1). AML patient samples were stored after informed consent at the HIMIP collection (BB-0033-00060). According to French law, HIMIP collections have been declared to the Ministry of Higher Education and Research (DC 2008-307 collection 1) and obtained a transfer agreement (AC 2008-129) after approbation by the “Comité de Protection des Personnes Sud-Ouest et Outremer II” (Ethical Committee). BUH and TUH cohorts are both registered in the Toulouse-Bordeaux DATAML registry [21, 22].

**Table 1:** Characteristics of patients from the TUH cohort.

2087 patients	TUH cohort	HSC-L	MPP-L	CMP-L	GMP-L	GP-L	MP-L
*Patients*							
Age (median and IQR, years)	65 (54–75)	61 (40–78)	69 (60–78)	69 (58–77)	62 (44–73)	69 (58–75)	63 (51–72)
<60 yr (%)	692 (33.2)	7 (38.9)	112 (24.5)	174 (27.6)	162 (45.0)	34 (28.6)	203 (40.4)
≥60 yr (%)	1395 (66.8)	11 (61.1)	346 (75.5)	456 (72.4)	198 (55.0)	85 (71.4)	299 (59.6)
*AML status*							
De novo	1423 (70.2)	13 (72.2)	243 (53.1)	400 (63.5)	276 (76.7)	96 (80.7)	395 (78.7)
Secondary to MDS	247 (12.2)	4 (22.2)	75 (16.4)	103 (16.3)	24 (6.7)	5 (4.2)	36 (7.2)
Secondary to MPN	142 (7.0)	1 (5.6)	70 (15.3)	51 (8.1)	12 (3.3)	3 (2.5)	5 (1.0)
Therapy-related	245 (12.1)	0 (0.0)	62 (13.5)	73 (11.6)	43 (11.9)	12 (10.1)	55 (11.0)
Unknown	30 (1.5)	0 (0.0)	8 (1.7)	3 (0.5)	5 (1.4)	3 (2.5)	11 (2.2)
*Extramedullary disease*							
Liver	100/1603	4/10	22/326	18/484	19/295	5/93	32/395
Spleen	161/1603	3/10	45/326	44/484	23/295	6/93	40/395
Lymph nodes	174/1603	4/10	26/326	37/484	32/295	10/93	65/395
Gingiva	148/1603	0/10	11/326	30/484	23/295	10/93	74/395
Skin	56/1603	3/10	3/326	14/484	10/295	1/93	25/395
*Complete Blood Count (median and IQR)*							
Hemoglobin (g/L)	9.3 (8.2–10.7)	8.9 (8.3–11.1)	9.0 (8.0–10.2)	9.3 (8.2–10.8)	9.4 (8.3–10.9)	9.4 (8.2–11.1)	9.6 (8.3–10.9)
Platelet count (g/L)	62 (33–112)	75 (37–163)	62 (29–120)	64 (31–115)	56 (32–101)	62 (34–98)	63 (39–110)
WBC count (g/L)	8.01 (2.40–36.79)	3.90 (1.40–12.76)	5.08 (2.05–20.20)	3.60 (1.80–15.53)	8.68 (2.88–44.98)	15.34 (6.9–80.10)	24.72 (4.30–71.36)
*Bone marrow evaluation*							
Blasts (median and IQR, %)	52 (30–77)	63 (39–90)	44 (29–69)	36 (24–61)	59 (37–76)	86 (62–93)	69 (44–86)
Cytological dysmyelopoiesis	912/1898	5/16	219/401	369/583	131/337	23/99	165/462
*Cytogenetic risk*							
Favorable (%)	139 (6.9)	0 (0.0)	0 (0.0)	15 (2.4)	119 (33.7)	1 (0.9)	4 (0.8)
Intermediate (%)	1258 (62.0)	9 (50.0)	221 (50.7)	343 (55.7)	165 (46.7)	103 (91.1)	417 (84.8)
Adverse (%)	631 (31.1)	9 (50.0)	215 (49.3)	258 (41.9)	69 (19.5)	9 (8.0)	71 (14.4)
*Mutation status (mutated/tested)*							
CEBPA mono or bi-allelic	69/865	0/6	1/163	9/248	41/125	4/59	14/264
DNMT3A (exon 23)	130/884	0/8	13/163	32/250	8/155	6/58	71/250
FLT3-ITD	295/1456	5/13	40/264	61/399	28/258	35/94	126/428
FLT3-TKD	57/818	1/6	8/159	15/234	8/152	4/55	21/212
IDH1	101/1120	0/8	10/231	29/325	12/193	13/72	37/291
IDH2	134/1120	1/8	25/231	45/325	23/193	17/72	23/291
NPM1	427/1421	0/9	16/271	49/394	18/232	75/94	269/421
*Treatment*							
Intensive chemotherapy (%)	1266 (60.7)	12 (66.7)	192 (41.9)	325 (51.6)	269 (74.7)	88 (73.9)	380 (75.7)
Allo-SCT (%)	299 (14.3)	3 (16.7)	69 (15.1)	88 (14.0)	50 (13.9)	11 (9.2)	78 (15.5)
Hypomethylating agents (%)	340 (16.3)	1 (5.6)	109 (23.8)	149 (23.7)	40 (11.1)	4 (3.4)	37 (7.4)
Best supportive care (%)	298 (14.3)	2 (11.1)	102 (22.3)	96 (15.2)	30 (8.3)	12 (10.1)	56 (11.2)
Other (%)	183 (8.7)	3 (16.7)	55 (12.0)	60 (9.5)	21 (5.8)	15 (12.6)	29 (5.8)

### Immunophenotyping

Multi-parameter flow cytometry (MFC) was performed on whole bone marrow (BM) or blood specimens using a standard stain-lyse-wash procedure with ammonium chloride lysis. 1 × 10^5^ cells were stained per analysis tube, and data were acquired on at least 1 × 10^4^ blasts when specimen quality permitted. Data on standardized 8- to 10-color staining combinations were acquired on FACSCanto II cytometers using FACSDiva software (BD Biosciences) or Navios instruments analyzed using Kaluza (Beckman-Coulter). Several different tube configurations were used through the course of the study, all with staining for CD13, CD33, CD34, CD45, CD117, HLA-DR, and cytoplasmic MPO. A blast gate including CD45 dim mononuclear cells was analyzed according to cytomorphologic data.

### Next-generation sequencing

DNA samples from 409 AML patients have been obtained after informed consent and stored at the HIMIP collection (BB-0033-00060). Briefly, genomic DNA was extracted from the baseline bone marrow sample using a Qiagen DNA extraction kit (Qiagen). The presence of *FLT3*-ITD was tested as described [19]. Electrophoregram peaks were quantified using GeneMarker 2.2 (SoftGenetics, State College, PA, USA). *CEBPA* screening was performed by classical Sanger sequencing [23]. An extended DNA resequencing was performed using a Illumina NextSeq500 and Sureselect (Agilent, Santa Clara, CA, USA) targeted on the complete coding regions of 46 genes commonly mutated in myeloid malignancies: *ASXL1* (NM_015338.6), *ASXL2* (NM_018263.6), *BCOR* (NM_001123383.1), *BCORL1* (NM_021946.5), *CBL* (NM_005188.4), *CCND2* (NM_001759.4), *CEBPA* (NM_004364.5), *CSF3R* (NM_156039.3), *DHX15* (NM_001358.3), *DNMT3A* (NM_022552.5), *EP300* (NM_001429.4), *ETV6* (NM_001987.5), *EZH2* (NM_004456.5), *FLT3* (NM_004119.3), *GATA1* (NM_002049.4), *GATA2* (NM_032638.5), *IDH1* (NM_005896.4), *IDH2* (NM_002168.4), *JAK2* (NM_004972.4), *KDM5A* (NM_001042603.3), *KDM6A* (NM_021140.4), *KIT* (NM_000222.3), *KMT2D* (NM_003482.4), *KRAS* (NM_004985.5), *MGA* (NM_001164273.2), *MYC* (NM_002467.6), *NF1* (NM_000267.3), *NPM1* (NM_002520.7), *NRAS* (NM_002524.5), *PHF6* (NM_032458.3), *PIGA* (NM_002641.4), *PTPN11* (NM_002834.5), *RAD21* (NM_006265.3), *RUNX1* (NM_001754.5), *SETBP1* (NM_015559.3), *SF3B1* (NM_012433.4), *SMC1A* (NM_006306.4), *SMC3* (NM_005445.4), *SRSF2* (NM_003016.4), *STAG2* (NM_001042749.2), *TET2* (NM_001127208.3), *TP53* (NM_000546.6), *U2AF1* (NM_006758.3), *WT1* (NM_024426.6), *ZBTB7A* (NM_015898.4), *ZRSR2* (NM_005089.4). Data were processed through two algorithms from GATK (https://software.broadinstitute.org/gatk), HaplotypeCaller, and Mutect2, and also through Agilent Surecall software, with a sensitivity of 1% [24, 25]. All variants called by two variant callers were checked using IGV software. Identified variants were curated manually and named according to the rules of the Human Genome Variation Society (hgvs.org).

### Bioinformatics analyses

Freely available gene expression datasets for normal hematopoietic stem and progenitor cells GEO:GSE42414 [26], Array Express:E-TABM-978 [12], GEO:GSE74246 [27], GEO:GSE63270 [28] were used for this study.

### Statistical analysis

Complete response and relapse rates were defined according to the Cheson criteria [29]. Comparisons were performed using a Mann–Whitney test or Kruskal–Wallis test for continuous variables and Fisher’s exact test for categorical variables with GraphPad Prism. Statistical test results are graphically expressed: *p < 0.05, **p < 0.01, ***p < 0.001.

Disease-free survival was measured from the date of complete remission until the date of relapse or death. The cumulative incidence of relapse was measured from the date of complete remission until the date of relapse, with death regarded as a competitive event. Overall survival was measured from the date of diagnosis until death. Patients in complete remission were censored at the time of the last contact. The risk groups for prognosis were evaluated for overall and disease-free survival by univariate analysis (log-rank test) using a multivariate model of Cox regression and for the cumulative incidence of relapse by the Fine and Gray test. All calculations were performed using STATA version 13 software (STATA Corp., College Station, TX, USA), all graphs were done using Graph Pad Prism.

More details are provided in Supplemental Information.

## Results

### Hematopoietic stem and progenitor cells (HSPC) immunophenotypes define AML specimens at different stages of the human hematopoietic hierarchy

**Fig. 1:**
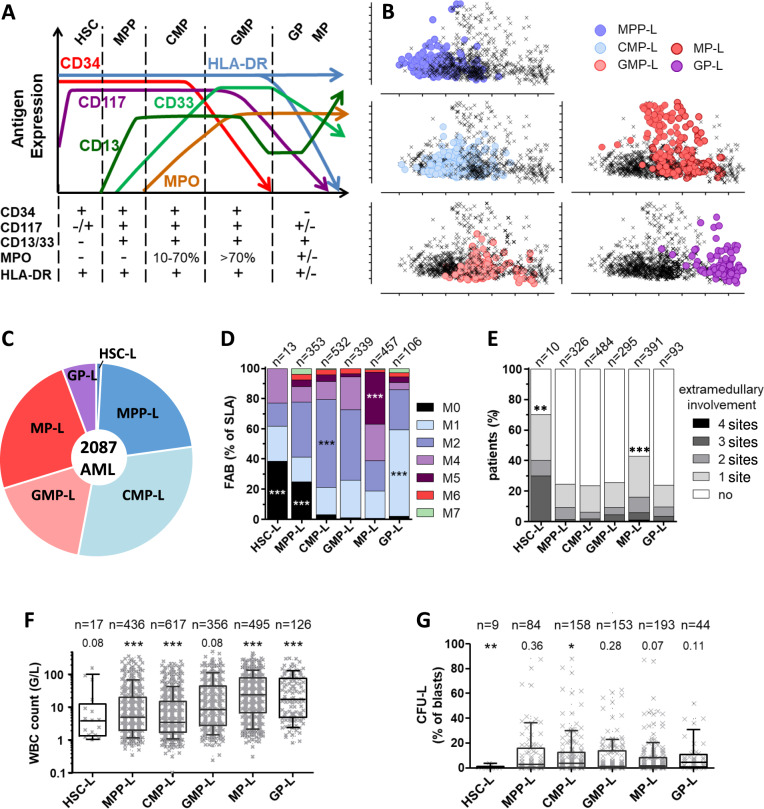
Phenotypic and clinical identification of AML subgroups. **A** Model of the relative percentage of myeloid marker expression over the course of normal HSPC differentiation. The SLA is defined by the combination of expressions of five myeloid markers plus HLA-DR to differentiate GP-L (HLA-DR+) and MP-L (HLA-DR−); +≥20% of blasts; −<20% of blasts; +/− marker can be positive or negative. **B** Principal component analyses of 945 AML using the percentage of AML blasts expression of 16 markers by flow cytometry (CD4, CD7, CD13, CD33, CD117, MPOc, CD34, HLA-DR, CD56, CD64, CD38, CD65, CD16, CD14, CD11b, CD123). AML patients were classified according to their SLA as detailed in (**A**). **C** Pie chart of 2087 AML from TUH cohort segregated according to their SLA. **D** FAB classification according to SLA in the TUH cohort. Fisher’s exact test compared FAB classification in one SLA to all others. **E** Extramedullary involvement in SLA (see Table 1 for details). **F** Boxplots of leukocytosis at diagnosis in TUH cohort. **G** CFU-L in TUH cohort. Statistical analysis was performed comparing one SLA to all others (Mann–Whitney test).

To define a surrogate phenotype for each stage of normal myeloid maturation, we need to assess by flow cytometry, the expression of markers used to characterize AML routinely on HSPCs. HSC, MPP, CMP, GMP, and GP/MP can be characterized by gating in the lineage negative cell population according to their expression of CD34, CD38, CD90, and CD45RA (Supplemental Fig. 1A, B). Unfortunately, the expression of those markers is not classically evaluated in AML. To overcome this issue, we evaluated the expression levels of four myeloid antigens (CD13, CD33, CD117, and myeloperoxidase [MPO]) as well as CD34 and HLA-DR in 10 normal human bone marrow samples. HSC and MPP were characterized by the absence of MPO expression (Fig. 1A and Supplemental Fig. 1C). The expression of specific myeloid markers such as CD13 or CD33 was detectable from the MPP stage onward. GMP displayed the highest MPO expression (>70% of cells). HLA-DR expression discriminated MP (HLA-DR positive) and GP (HLA-DR negative). These data show that six markers (i.e, CD34, CD117, CD13, CD33, MPO, and HLA-DR) used to diagnose AML in routine clinical practice are differentially expressed in six immunophenotypically defined stages of normal myelopoiesis.

To correlate each stage of normal hematopoiesis with that of individual AML, we transformed HSPC immunophenotypes into immunophenotypic AML signatures (Fig. 1A). Thus, the SLA was assigned to each sample, based on leukemic bulk phenotype, because in the hierarchy of leukemic cells, the majority are stopped in their differentiation pathway. We tested our six-marker immunophenotypic signature by looking at the principal component analysis distribution of 945 AML, extensively characterized by the expression of 16 antigens (Fig. 1B and Supplemental Fig. 2A and B). Our six-marker signature was sufficient to discriminate between different hematopoietic/leukemic groups (Supplemental Fig. 2C, D). Overall, we defined, by flow cytometry, the stage at which the leukemic cell population accumulated as the stage of leukemia differentiation arrest.

Our data show that AML with an immunophenotypic signature similar to that of HSC (henceforth termed HSC-L) represented 0.9% (Fig. 1C). TUH cohort comprised 21.9% of MPP-L, 30.2% of CMP-L, 17.2% of GMP-L, 24.1% of MP-L, and 5.7% of GP-L. HSC-L and MPP-L were enriched in AML classified as FAB M0, while MP-L was enriched in acute monoblastic leukemia (FAB M5) and GP-L in AML classified as FAB M1 (Fig. 1D). The FAB M6 and M7 represented 2.9% and 1.4% of the TUH cohort, respectively. Classified by phenotype, their SLA were heterogeneous (Fig. 1D). Nevertheless, the SLA of 63.3% of the FAB M6 and M7 was identified as MPP-L or CMP-L, stages prior to the MEP branch (Supplementary Fig. 1A), whereas the remainder (1.5% of the total cohort) may have been misclassified because of the absence of erythroid and megakaryocytic markers in our panel. Thus, flow-based analysis of SLA correlates with the morphologic phenotype used to assign the FAB sub-group although the correlation is not completely consistent.

### SLA retain functional and genetic imprints of their normal counterparts

Following induction of the differentiation process, HSPCs lose their capacity to self-renew, in favor of proliferation and migration. We, therefore, investigated the functional characteristics of the six SLA using clinical data and clonogenic properties as surrogate markers of migration (extramedullary involvement) and proliferation (leucocytosis) capacities. Cell migration capacities and emigration from the bone marrow are known to be features acquired during differentiation [30]. As a result, the extramedullary disease was significantly more frequently observed in the MP-L group and surprisingly in the low HSC-L group (Fig. 1E). In detail, patients with GMP-L, GP-L, and MP-L displayed a higher rate of lymph node enlargement and leukemic gingival infiltration (Table 1). However, spleen enlargement was mostly seen in MPP-L. Interestingly, leucocytosis increased as SLA was further advanced in the differentiation process (Fig. 1F and Table 1). Moreover, the clonogenic capacities of the HSC-L, similar to normal HSC [31], were significantly lower than those of other SLA (Fig. 1G).

**Fig. 2:**
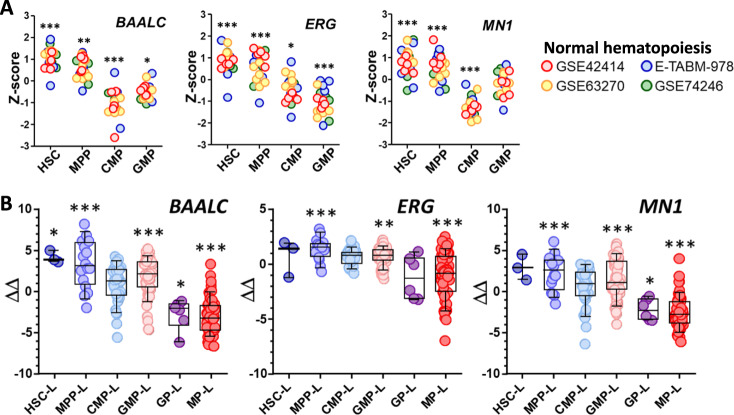
Genetic validation of SLA classification. **A** Expression of *BAALC*, *ERG*, and *MN1* in four normal HSPCs datasets. Gene expressions were normalized calculating *Z*-score in each dataset. **B** Expression of *BAALC*, *ERG*, and *MN1* according to SLA subgroups in TUH cohort (fluidigm, *n* = 171).

Since the SLA is defined by HSPCs phenotypes, we hypothesized that the expression of genes known to be expressed in AML could be related to the SLA. To test our hypothesis, we focus on well-described AML prognostic genes *BAALC*, *ERG*, and *MN1* [32, 33] and analyzed the publicly available transcriptomic HSPCs database. Those three genes were overexpressed in HSC and their expression level decreased as hematopoietic differentiation progressed (Fig. 2A). Similarly, *BAALC*, *ERG*, and *MN1* were overexpressed in HSC-L and MPP-L and repressed in GP-L and MP-L (Fig. 2B).

Therefore, we showed in the TUH cohort that the different SLAs retained specific biological characteristics of normal hematopoiesis.

### SLA correlates with leukemic stem cell profiles of AML

**Fig. 3:**
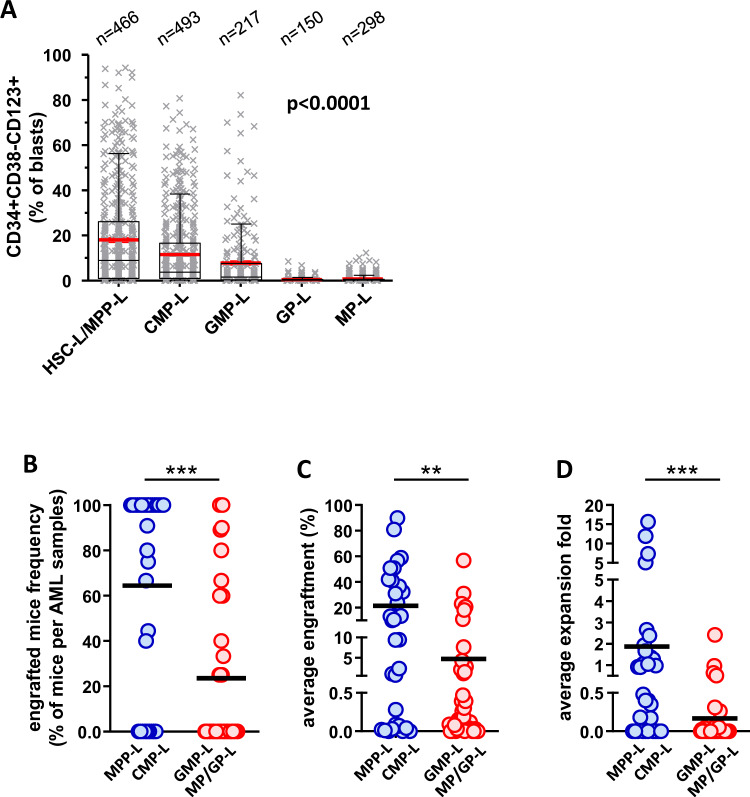
Stem cell properties are related to the SLA. **A** Percentage of leukemic stem cells (CD34^+^CD38^−^CD123^+^) among blasts according to SLA in the TUH cohort (Kruskal–Wallis test). **B**–**D** Patient-derived xenograft from 70 AML patient samples in 446 mice. A group of five mice is classically used to test an AML sample with an injected dose of 10^7^ cells per mouse. Engraftment is assessed in a delay of 16 weeks. **B** Percentage of mice with >0.5% of human leukemic cells detected in bone marrow samples by flow cytometry. **C** Evaluation of human leukemic engraftment in bone marrow samples of each experiment. Each point represents the mean of all PDX of a donor. **D** Expansion fold is calculated as a ratio between engrafted cells in mice bone marrow and spleen and injected leukemic cells.

We have previously shown that the level of CD34^+^CD38^−^CD123^+^ LSCs is an independent prognostic factor in AML treated with intensive chemotherapy [16, 34]. To study the relationship between SLA and LSC, we measured LSC levels in the TUH cohort (Fig. 3A). HSC-L/MPP-L had the highest levels of CD34^+^CD38^−^CD123^+^ LSCs (18.03% vs. 11.54% in CMP-L, 7.83% in GMP-L, <1% in MP-L and GP-L, Kruskal–Wallis test *p* < 0.0001).

To evaluate the stem properties of SLA subsets, we injected leukemic cells from 70 AML in 446 NGS mice (6.4 mice/sample, range 4–20). Early SLA (i.e., MPP-L and CMP-L, 31 AML, 209 mice) had higher number of engrafted mice (64.4% vs. 23.5%, *p* = 0.0001, Fig. 3B), higher levels of engraftment (21.5% vs. 4.7%, *p* = 0.0027, Fig. 3C), and greater expansion of leukemic cells (1.9 vs. 0.2-fold, *p* = 0.0002, Fig. 3D) than late SLA (GMP-L, MP-L, and GP-L, 38 AML, 237 mice).

Together, those data show that stem properties are enriched in early SLA (HSC-L, MPP-L, and CMP-L).

### Oncogenic events are specific to SLA

**Fig. 4:**
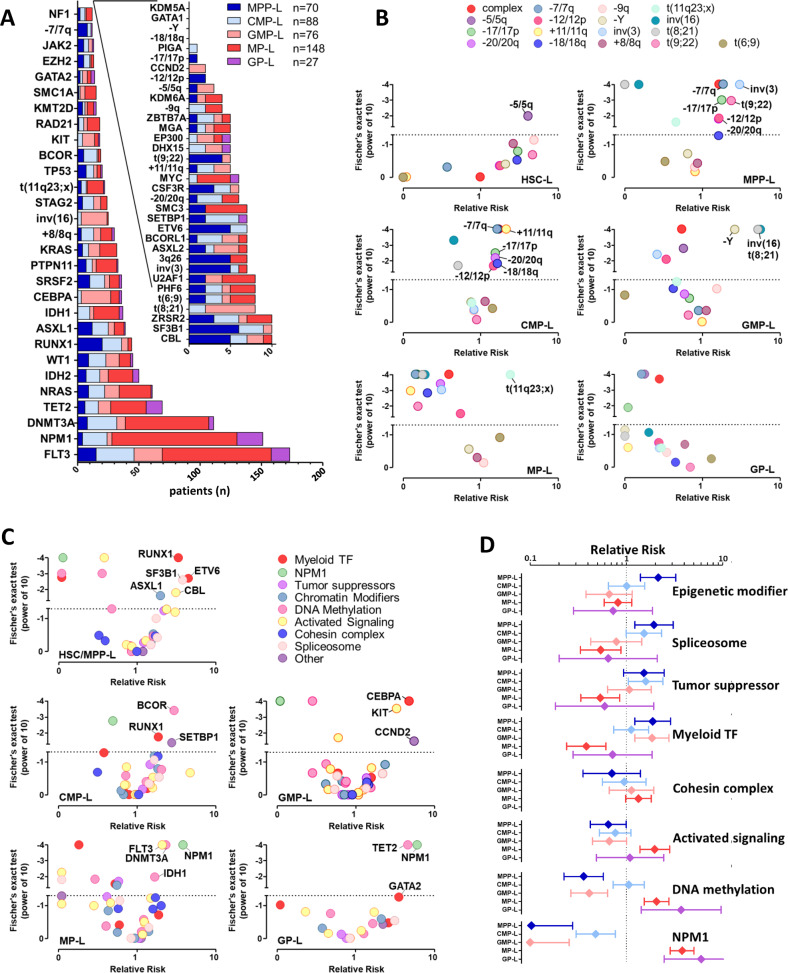
Distribution of AML mutations and genetic abnormalities according to the SLA. **A** Number of patients with specific mutations or genetic abnormalities (*n* = 409). **B** Volcano plots of relative risk of the presence of specific genetic anomalies in each SLA (*n* = 1967). **C** Volcano plots of relative risk of the presence of specific mutations in each SLA (*n* = 409). **D** Plots of relative risks of eight functional modules of mutations [35] in SLA.

To identify oncogenic events linked to specific SLA, we studied point mutations and cytogenetic anomalies. We screened 46 genes commonly mutated in myeloid malignancies from 409 patients of the TUH cohort and identified 1363 mutations or cytogenetic anomalies (Fig. 4A), with overall frequencies that were consistent with those published in previous studies [35, 36]. We identified at least one driver mutation in 399 patients (97.6%) and two or more driver mutations in 89.7% of the samples.

Although co-mutation or mutual exclusivity profiles have been previously described in AML [35, 36], our cohort allowed a more comprehensive analysis of the driver mutations involved in the maturation block of SLA. We calculated the relative risks (RR) of cytogenetic abnormalities (*n* = 1967, Fig. 4B) and point mutations (*n* = 409, Fig. 4C) for each SLA.

### MPP-L and CMP-L show criteria of secondary AML

**Fig. 5:**
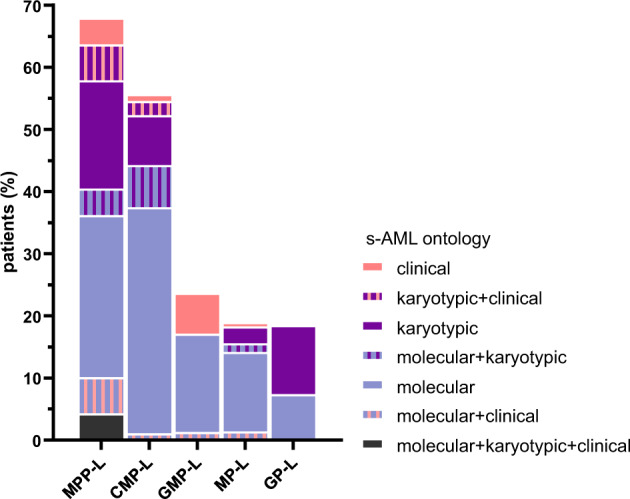
Secondary AML according to the SLA. Definition of secondary AML in 409 patients based on an association of clinical (history of MDS or MPN), molecular (mutations in any of the eight genes frequently altered in MDS [37]) and/or karyotypic abnormalities as defined by WHO.

MPP-L and CMP-L are phenotypically defined as CD34^+^ AML, positive for myeloid markers (CD13^+^CD33^+^CD117^+^); and differ by their expression of cytoplasmic MPO (<10% for MPP-L and within the range of 10–70% for CMP-L). MPP-L and CMP-L show more often cytogenetic abnormalities of AML MRC (Fig. 4B) such as del(7q) (RR:1.85, *p* < 0.0001; RR:1.61, *p* < 0.0001; for MPP-L and CMP-L, respectively), del(17p) (RR:1.77, *p* = 0.0010; RR:1.53, *p* = 0.0033, respectively) and del(12p) (RR:1.63, *p* = 0.015; RR:1.46, *p* = 0.021, respectively). MPP-L and CMP-L are also enriched in secondary AML (s-AML) mutations [37] in normal karyotype (*n* = 200, Supplemental Fig. 3A, B): *ASXL1* (MPP-L RR:6.1; *p* = 0.0006), *SRSF2* (MPP-L RR:5.0; *p* = 0.0021), *EZH2* (MPP-L RR:7.7; *p* = 0.024), *ZRSR2* (MPP-L RR:5.8; *p* = 0.046), *STAG2* (MPP-L RR:2.9; *p* = 0.092), and *SF3B1* (CMP-L RR:3.3; *p* = 0.050), *BCOR* (CMP-L RR:2.7; *p* = 0.089). In order to investigate the relationship between secondary AML and SLA, we rigorously classified 409 AML patients as clinical s-AML (post-MDS or MPN), molecular s-AML (defined as AML with mutations in any of the eight genes frequently altered in MDS [37]) or karyotypic s-AML (Fig. 5). MPP-L and CMP-L were classified s-AML in 68% and 56%, respectively (RR:3.0, *p* < 0.0001). In addition, inv(3) (RR:3.0, *p* < 0.0001), t(9;22) (RR:2.4, *p* = 0.0011), *CSF3R* (normal karyotype RR:12.3, *p* < 0.0001) and *RUNX1* mutations (RR:3.3, *p* < 0.0001) were enriched in MPP-L.

Gene mutations can be further functionally classified into eight categories [35] (Fig. 4D and Supplemental Fig. 3C). MPP-L were enriched in mutations in epigenetic modifiers (RR: 2.1, *p* = 0.001), spliceosome (RR:1.9, *p* = 0.01) and myeloid transcription factors (mainly *RUNX1* and *ETV6* mutations, RR:1.9, *p* = 0.008).

### *Bi-allelic CEBPA* mutations and CBF abnormalities are specific of GMP-L

GMP-L is defined with a classic phenotype CD34^+^CD13^+^CD33^+^CD117^+^ and high expression of cytoplasmic MPO (>70%). Astonishingly, it was very specific of three abnormalities (Fig. 4B, C): inv(16) (RR:5.6, *p* < 0.0001), t(8;21) (RR:5.2, *p* < 0.0001) and *CEBPA* mutations (RR:4.8, *p* < 0.0001). We further studied *CEBPA* mutations in 871 AML from the TUH cohort and found the mutation in 35.7% of GMP-L (46/129, RR:6.2, *p* < 0.0001, Supplemental Fig. 4A), the majority of which were bi-allelic mutations (72%, 33/46). Overall, CBF abnormalities represented 33% of GMP-L (119/360 patients).

### MP-L and GP-L are the two sides of *NPM1* mutated AML

MP-L and GP-L are phenotypically defined as CD34^−^ AML, positive for myeloid markers (CD13^+^CD33^+^CD117^+/−^); and differ by their expression of HLA-DR (≥20% for MP-L and <20% for GP-L). Both groups frequently expressed *NPM1* mutation (MP-L RR:3.8, *p* < 0.0001; GP-L RR:6.0, *p* < 0.0001, Fig. 4C). However, *NPM1* mutations were associated with mutations of *DNMT3A* (RR:2.3, *p* < 0.0001) and *FLT3* (RR:2.1, *p* < 0.0001) in MP-L, and with *TET2* mutations in GP-L, (RR:4.6, *p* < 0.0001). Mutations in *TET2*, *IDH1*, and *IDH2* are largely mutually exclusive and lead to similar epigenetic changes [38]. Since the *TET2* mutations were enriched in GP-L, we looked at the distribution of *IDH1* and *IDH2* mutations in the TUH cohort and found that these mutations were also enriched in GP-L (Supplemental Fig. 4B). Indeed, the GP-L subgroup was composed of *NPM1*/*TET2* mutated and *NPM1/IDH1* or *NPM1/IDH2* mutated patients (52% and 20% of GP-L, respectively). Of note, besides the *NPM1*-mutated MP-L subset which accounts for 64% of all MP-L and 82% of normal karyotype MP-L, *MLL* fusions were enriched in this SLA (RR:2.4, *p* < 0.0001; Fig. 4B) although their frequency is modest (59 patients in TUH cohort including 32 MP-L).

### SLA correlates by chemoresistance and outcome of patients treated by intensive chemotherapy

**Fig. 6:**
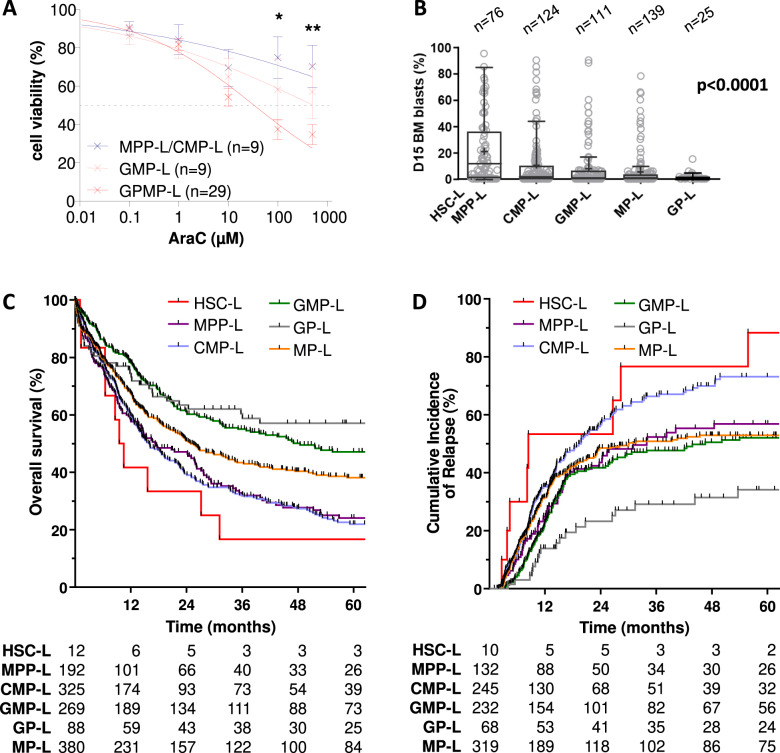
Response to chemotherapy according to the SLA. **A** In vitro testing of cytarabine (AraC) activity in 47 AML samples. **B** Early chemosensitivity according to SLA was evaluated in patients by measuring the percentage of residual blasts in bone marrow at day 15 of induction chemotherapy (*n* = 475). **C** Prognostic impact of SLA on overall survival for patients from TUH cohort treated with intensive chemotherapy (*n* = 1266). See Table S2 for multivariate analysis results. **D** Prognostic impact of SLA on overall survival for younger patients (<60 years) from TUH cohort treated with intensive chemotherapy (*n* = 638).

We investigated ex-vivo chemosensitivity and the response to intensive chemotherapy of AML patients according to their SLA. Ex-vivo apoptosis testing of 47 AML samples incubated with cytarabine (AraC) showed that MPP-L and CMP-L had a significantly higher IC_50_ than GMP-L and GP/MP-L (>1000 vs. 540 and 33 μM, respectively, Fig. 6A). Moreover, AML patients with immature SLA had a higher percentage of residual blasts in bone marrow at day 15 after intensive chemotherapy (Fig. 6B) and consequently, a lower complete response rate than patients with more mature SLA (HSC/MPP-L 72%; CMP-L 76%; GMP-L 87%; MP-L 85%; GP-L 79%; *p* < 0.0001). As a result, overall survival was significantly worse in patients with immature compared to mature SLA (*p* < 0.0001, Fig. 6C and Supplemental Fig. 5A) even though the early death rate was higher in hyperleukocytic SLA (GP-L and MP-L, Table S2). The cumulative incidence of relapse (CIR) was also significantly higher in the immature SLA group (*p* < 0.0001, Fig. 6D and Supplemental Fig. 6A). The correlation between SLA and response to chemotherapy was confirmed in younger AML patients (Supplemental Figs. 5B and 6B). Consistent with their chemoresistance status, allogeneic stem cell transplant in first complete remission was of great survival benefit for MPP-L and CMP-L and showed little or no survival improvement in the other groups (Table S3). Interestingly, SLA of relapsed AML (*n* = 193) was identical or more immature to diagnostic, in most of the cases (57% and 27%, respectively, Table S4). When a more mature SLA was identified at relapse (16%, 30/193), we observed, when available, a modification of the mutational profile in half of the cases (8/16).

In multivariate models, SLA classification retained independent prognostic values for overall survival, event-free survival, and cumulative incidence of relapse (Tables S5 and S6). Of note, GP-L represented a good prognostic subgroup, with a plateau of CIR at 37% in the TUH cohort (Supplemental Fig. 6A) and 19% in those under 60 (Supplemental Fig. 6B).

Altogether, these data indicate that the chemoresistance of AML cells is, at least in part, a consequence of innate (SLA imprint) and acquired (oncogenic events) mechanisms (see Table S7 for the summary of characteristics of SLA).

### Validation in an independent cohort of 1209 AML patients

To robustly validate our signatures, we took advantage of a second AML cohort: 1209 patients diagnosed at Bordeaux University Hospital (BUH cohort, see Table S1). Similarly, to the TUH cohort, the BUH cohort comprised 0.7% of HSC-L, 11.7% of MPP-L, 27.9% of CMP-L, 28.4% of GMP-L, 21.9% of MP-L, and 9.4% of GP-L (Supplemental Fig. 7A). HSC-L and MPP-L were enriched in AML classified as FAB M0, while MP-L were enriched in acute monoblastic leukemia (FAB M5) and GP-L in AML classified as FAB M1 (Supplemental Fig. 7B). Leukocytosis increased as SLA was further advanced in the differentiation process (Supplemental Fig. 7C).

In the BUH cohort, we found that inv(3) (Supplemental Fig. 8A) were enriched in MPP-L, whereas ASXL1 mutation and t(9;22) were increased but not statistically specific to this SLA (Supplemental Fig. 8B, C); inv(16), t(8;21) and bi-allelic *CEBPA* mutations were enriched in GMP-L (Supplemental Fig. 8D, E); *MLL* fusions and *NPM1* and *DNMT3A* mutations were enriched in MP-L (Supplemental Fig. 8F–H) whereas *NPM1* and *TET2* and *IDH* mutations were enriched in GP-L (Supplemental Fig. 8I–K).

In the BUH cohort, SLA retained their prognostic factor, with increased D15 blasts (Supplemental Fig. 9A), and worse OS and CIR (Supplemental Fig. 9B, C) in immature SLA.

## Discussion

It has long been possible to immunophenotypically classify acute lymphoblastic leukemia [39–41]. These classifications are based on the expression by normal lymphocytes of antigens that are specific for different maturation stages. To date, such classifications do not apply to leukemia of the myeloid lineage likely because human myelopoiesis is less strictly defined than lymphopoiesis and is regularly reconsidered [3, 4, 12, 42–45]. Here, we presented a phenogenomic framework of AML that provides insight into the pathogenesis of AML and that identifies molecular features influencing therapy response. We discovered five distinct phenotypic subgroups that differ by specific surface protein expression patterns and hence provide a phenotypic classification of AML. Our study builds on previous work that cataloged genetic aberrations in AML and linked them to clinical outcomes, resulting in a genomic classification of the disease [36, 46]. We showed an exclusive association between a few genomic alterations and hematopoietic maturation stages. Interestingly, previous transcriptomic studies found at least 16 AML subgroups that were also associated with specific cytogenetic features and mutations [47]. Of these, only the GMP-L-associated genomic aberrations (CEBPAm, t(8;21) and inv16) were directly associated with transcriptomic clusters. Altogether, this suggests that, in most cases, genomic, transcriptomic, and proteomic data are independent and complementary.

The clinical relevance of our AML proteomic classification is further supported by the fact that proteomic clusters significantly differed in their outcomes in patients treated with intensive chemotherapy. Moreover, complementary to morphological analysis, we believe that this classification which can be available on the day of diagnosis, whereas cytogenetic and molecular abnormalities are available only a few days later or sometimes missing, may inform physicians on the disease subtype and contribute to patient management (Supplemental Fig. 10).

Although the SLA classification clearly segregated HSC-L/MPP-L from GMP-L and GP-L/MP-L, the CMP-L subgroup was more heterogeneous. This may suggest that this stage is insufficiently characterized by its immunophenotypic signature and/or that its normal counterpart is itself heterogeneous and should be separated into several more homogeneous stages. Alternately, complex mechanisms of differentiation arrest could apply to this subtype in which no recurrent genetic events were identified at variance with HSC-L, MPP-L, GMP-L, GP-L, and MP-L. More studies are needed to identify new surface markers in order to refine the SLA classification which may encompass more groups.

Our findings also suggest that few oncogenic events may be responsible for the SLA. *RUNX1* mutations and inv(3) are associated with MPP-L, abnormalities of secondary AML with MPP-L and CMP-L, *CEBPA* mutations*, RUNX1-RUNX1T1* or *CBFB-MYH11* translocations with GMP-L, *NPM1*, and *TET2* or *IDH* mutations with GP-L and *NPM1* and *DNMT3A* mutations and t(11q23) with MP-L. As noted, future studies will need to further clarify the genotype–phenotype correlations of AML as improved myeloid maturation markers are developed.

The subclonal architecture of AML has been already described [48]. Hypothetically, the presence of different leukemic clones, blocked at different stages, could interfere with the SLA signature determination. Although we cannot completely exclude this possibility, some elements argue in favor of a weak impact of subclonal architecture on SLA signatures: (i) most AML at the time of diagnosis are composed of a major founding clone likely to be detected by the SLA signature [35, 48]; (ii) oncogenic mutations strongly linked to SLA are rarely found in pre-leukemic clones (except DNMT3A or TET2 mutations) but are likely a later event in leukemogenesis [49, 50]; and consequently (iii) these oncogenic events are mutually exclusive and rarely found associated in AML patients. Nevertheless, the molecular complexity of CMP-L raises the question of the subclonal architecture of this subtype. Further genetic and immunophenotypic studies are needed to fully explore the relationship between SLA and AML architecture as some leukemic clones show functional heterogeneity [51].

In vitro studies of chemosensitivity and clinical data also demonstrated that the SLA classification could predict response to the main therapeutic strategy used in AML. Indeed, GMP-L/GP-L/MP-L encompass the more chemosensitive genetic subgroups (i.e, *RUNX1-RUNX1T1*, *CBFB-MYH11*, *CEBPA*, and *NPM1* mutations) do benefit from intensive chemotherapy as compared with HSC-L/MPP-L/CMP-L. Obviously, it will be very interesting to describe the impact of new therapeutic combinations such as azacitidine and venetoclax in this context [52]. Furthermore, this SLA classification is a useful tool in clinical practice because it may predict on the day of diagnosis in which genetic subgroup patients will be ultimately classified by chromosomal and molecular analyses. This may have an impact on clinical management. Furthermore, this correlation may suggest that AMLs that are more closely related to HSPC are most likely to retain the chemoresistance properties of HSCs. Studies of the phenotype of residual leukemic cells after chemotherapy induction may help clarify the biology of chemoresistance.

In summary, AML immunophenotyping can establish a new SLA classification that strongly correlates with cellular behavior of the leukemic bulk, and predicts main genetic subgroups early at diagnosis and outcome after intensive chemotherapy. Each SLA is defined by specific oncogenic events whose penetrance may be dependent on the differentiation stages of hematopoiesis and their gene expression. Identifying disrupted gene pathways specific for each SLA should therefore form the basis for targeted therapies aimed at inducing AML differentiation.

## Supplementary information

Supplemental material and mlethods annd Figures

Supplemental Table 1

Early deaths assessment in SLA.

Landscape analysis of allografted and non-allografted AML (<65 years) in CR according to SLA.

Comparison of SLA at diagnosis and at relapse

Cox regression model for overall survival of TUH cohort (related to Figure 6).

Competing risk regression model for cumulative incidence of relapse of TUH cohort (related to Figure 6).

Summary of AML patient characteristics according to the SLA classification.

Cox regression model for overall survival of BUH cohort (related to Supplemental Figure 7).

## Data Availability

The datasets generated during and/or analyzed during the current study are available from the corresponding authors on reasonable request.
