# Supplementary material for: Phenotypically-defined stages of leukemia arrest predict main driver mutations subgroups, and outcome in acute myeloid leukemia
Source: Blood Cancer J. 2022 Aug 16;12(8):117. doi: 10.1038/s41408-022-00712-7 (PMC9381519; doi:10.1038/s41408-022-00712-7)
Supplement: Supplementary file 1 — Supplemental material and mlethods annd Figures [file 41408_2022_712_MOESM1_ESM.docx]

**Phenotypically-defined stages of leukemia arrest predict main driver mutations subgroups, and outcome in acute myeloid leukemia**

François Vergez et al.

**SUPPLEMENTAL METHODS**

**Immunophenotyping.** HSPCs were obtained from healthy donors after informed consent. Mononuclear cells were isolated using density gradient centrifugation then by collecting cells enriched in CD34+, using immunomagnetic beads (Miltenyi Biotech, Bergisch Gladbach, Germany). The distinction between HSPC sub-populations was achieved by labelling cells with a fluorochrome-conjugated antibody cocktail directed against lineage markers (Lin includes CD2, CD3, CD4, CD7, CD8, CD10, CD11b, CD14, CD19, CD20, CD56 and CD235a) as well as with antibodies targeting CD34, CD38, CD90, CD45RA and CD133 (BD Biosciences, Franklin Lakes, NJ). Flow cytometry analysis was carried out on a Navios instrument with Kaluza software (Beckman Coulter, Brea, CA). Analysis of the leukemic blasts took into account cells positive for the membrane markers CD34, CD117, CD13, CD33, HLA-DR and cytoplasmic myeloperoxidase (MPO).

**Patient-derived xenografts.** Primary AML cells were injected the tail vein of NSG mice in 100-200 μL of PBS at a concentration of 50 × 10^6^/mL. Primary leukemia engraftment was defined as >1% human CD45^+^ cells in the peripheral blood by flow cytometry. Mice were sacrificed according to protocol no later than 16 weeks or when moribund or upon development of hind-limb paralysis**.** All *in vivo* procedures were conducted in accordance to a protocol approved by the UPenn Institutionnal Animal Care and Use Committee (IACUC).

**Cell Survival and Colony Assays.** AML CD45low SSClow cell survival was determined by annexin V-FITC (BD Biosciences) and 7-AAD (Sigma-Aldrich, Allentown, PA) negativity after 24h culture with or without cytarabine (AraC) in IMDM 10% fetal calf serum (Sigma-Aldrich). Enumeration was performed by a FACS Canto II (BD Biosciences) or a Navios (Beckman-Coulter) instrument. For colony assays, AML cells from 641 patients at diagnosis were adjusted to a final concentration of 1x10^5^ cells/mL and grown in H4230 Stem Cell Technologies methyl cellulose medium (Stem Cell Technologies, Vancouver, Canada) supplemented with 10% 5637-conditioned medium ^20^. Leukemic colonies (more than 20 cells) and clusters (more than 5 cells) were counted at day 7 and uniformly named CFU-L (Colony Forming Unit-Leukemia). When needed, the viability and leukemic nature of CFU-L were confirmed by a blue trypan exclusion assay and by morphological analysis after Giemsa staining.

**Gene expression.** Expression levels of *BAALC* (Hs00227249_m1), *ERG* (Hs01554635_m1) and *MN1* (Hs00159202_m1) in 171 AML samples were analyzed using a Biomark 96.96 Dynamic Array platform (Fluidigm, South San Francisco, CA) according to the manufacturer’s instructions. Data obtained from the BioMark Realtime PCR Analysis V2.1.1 software were normalized according to the expression of four of the most stable housekeeping genes (*GAPDH*, *GUSB*, *TBP* and *ABL1*, GeNorm algorithm) and expressed as ΔΔCt.

**SUPPLEMENTAL FIGURES AND FIGURE LEGENDS**


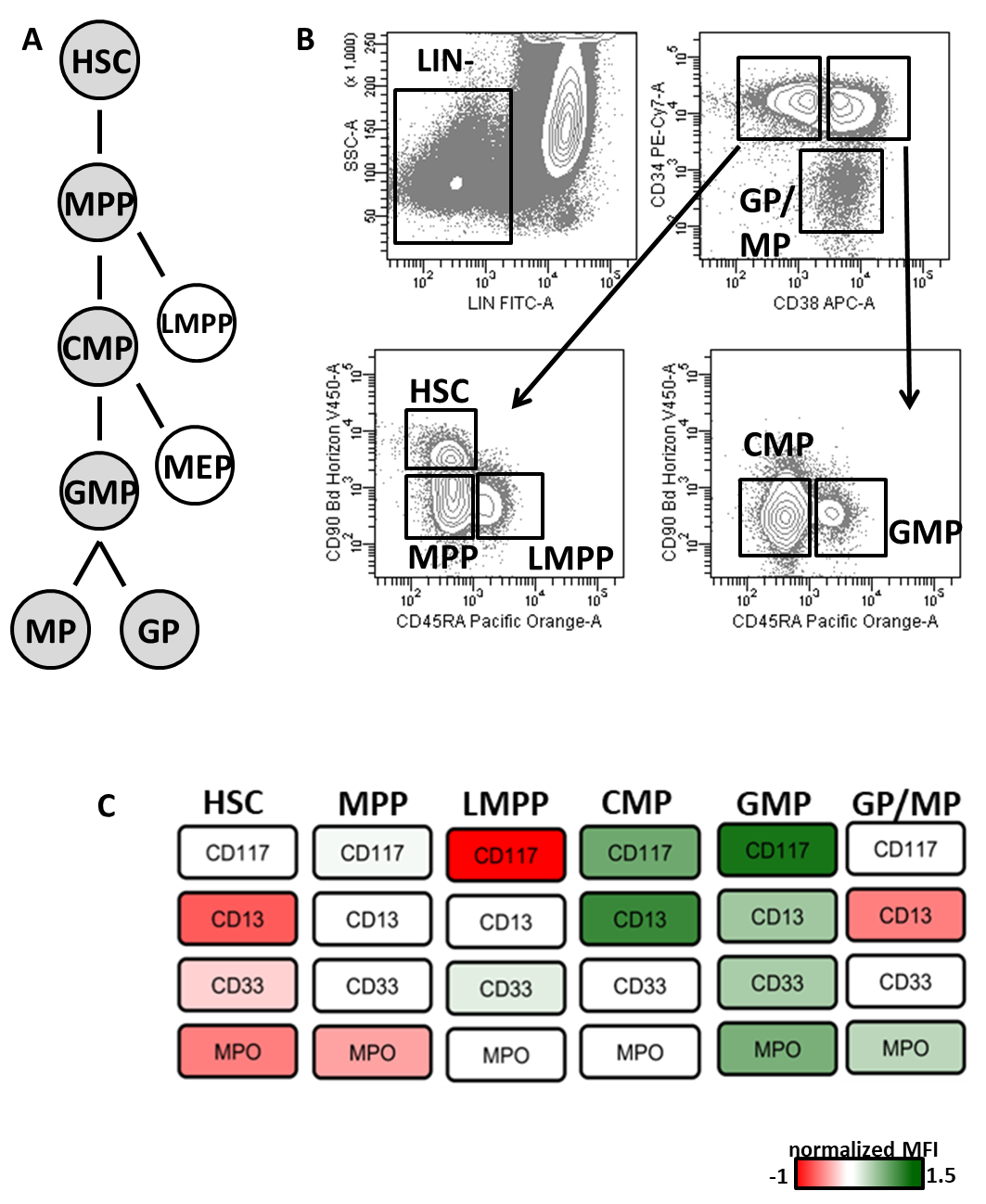


***Supplemental Figure 1: Phenotypic identification of normal hematopoietic stem and progenitors subgroups (related to Figure 1)***. (A) Model of hierarchical differentiation of hematopoietic stem and progenitor cells use in this work. HSC: Hematopoietic Stem Cell; MPP: Multi-Potent Progenitor; CMP: Common Myeloid Progenitor; GMP: Granulocyte-Monocyte Progenitor; GP: Granulocyte Progenitor; MP: Monocyte Progenitor; LMPP: Lymphoid-primed Multi-Potent Progenitor; MEP: Megacaryocyte-Erythroid Progenitor. (B) Gating strategy to characterize the expression of AML markers in normal HSCPs from seven healthy donors. (B) Representation of the normalised mean fluorescence intensity of the four myeloid antigens tested (CD117, CD13, CD33 and MPO) over the course of physiological hematopoiesis. Each rectangle represents the average result (n=7).


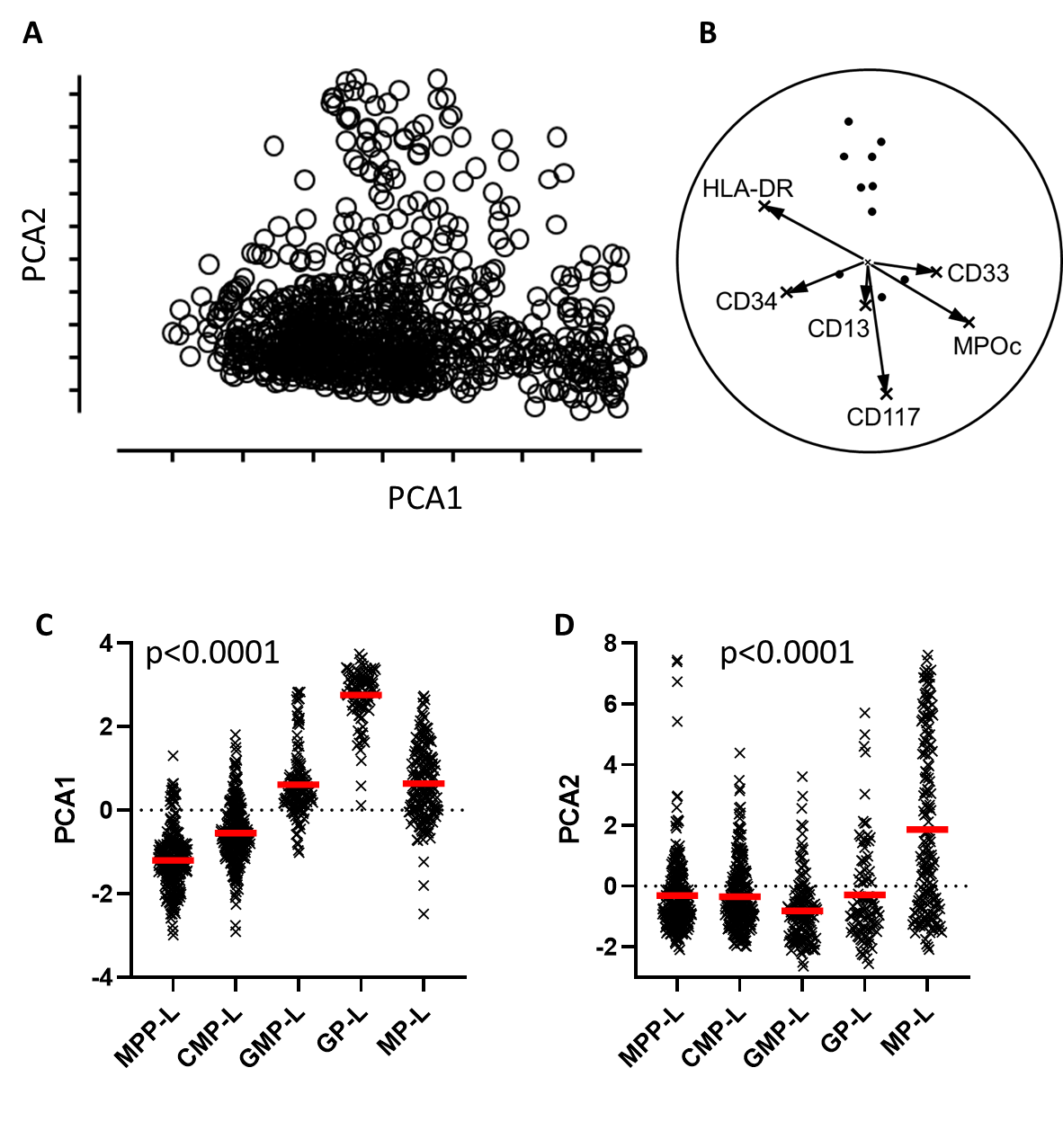


***Supplemental Figure 2: Unsupervised principal component analysis of 945 AML (related to Figure 1).*** (A) Dot plot of 945 AML calculated according to the expression of 16 phenotypic markers by leukemic blasts in principal component analyses. (B) Vectorial presentation of the 16 parameters included in the analysis. Phenotypic makers that have been chosen for the study are symbolized by an arrow. (C) PCA1 values of each SLA. (D) PCA2 values of each SLA.


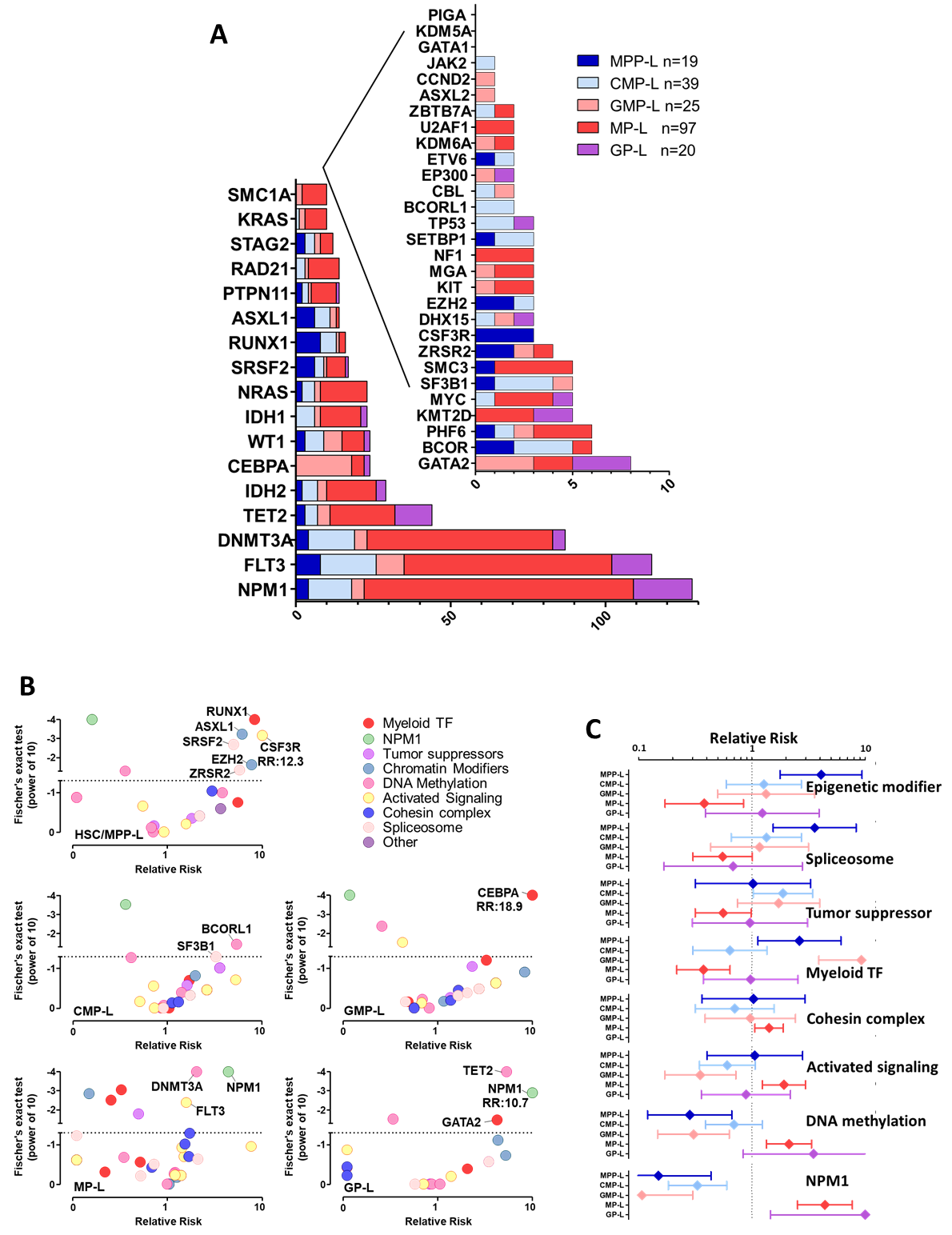


***Supplemental Figure 3: Distribution of mutations in AML with normal karyotype according to the SLA (related to Figure 4).*** (A) Number of patients with specific mutations or genetic anomalies (n=200). (B) Volcano plots of relative risk of the presence of specific mutations in SLA (n=200). (C) Plots of relative risks of 8 functional modules of mutations^25^ in SLA.


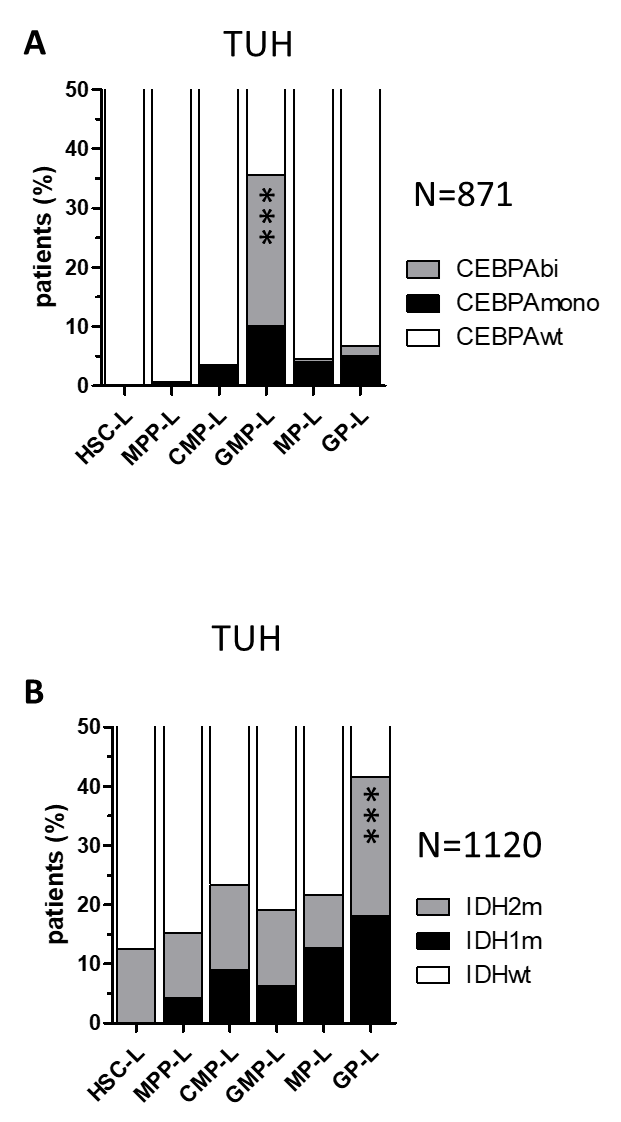


***Supplemental Figure 4: Validation of SLA specific mutations and genetic anomalies in TUH cohort (relative to Figure 5).*** (A) Percentage of *CEBPA* mutations according to SLA (n=871). (B) Percentage *IDH* mutations according to SLA (n=1120).


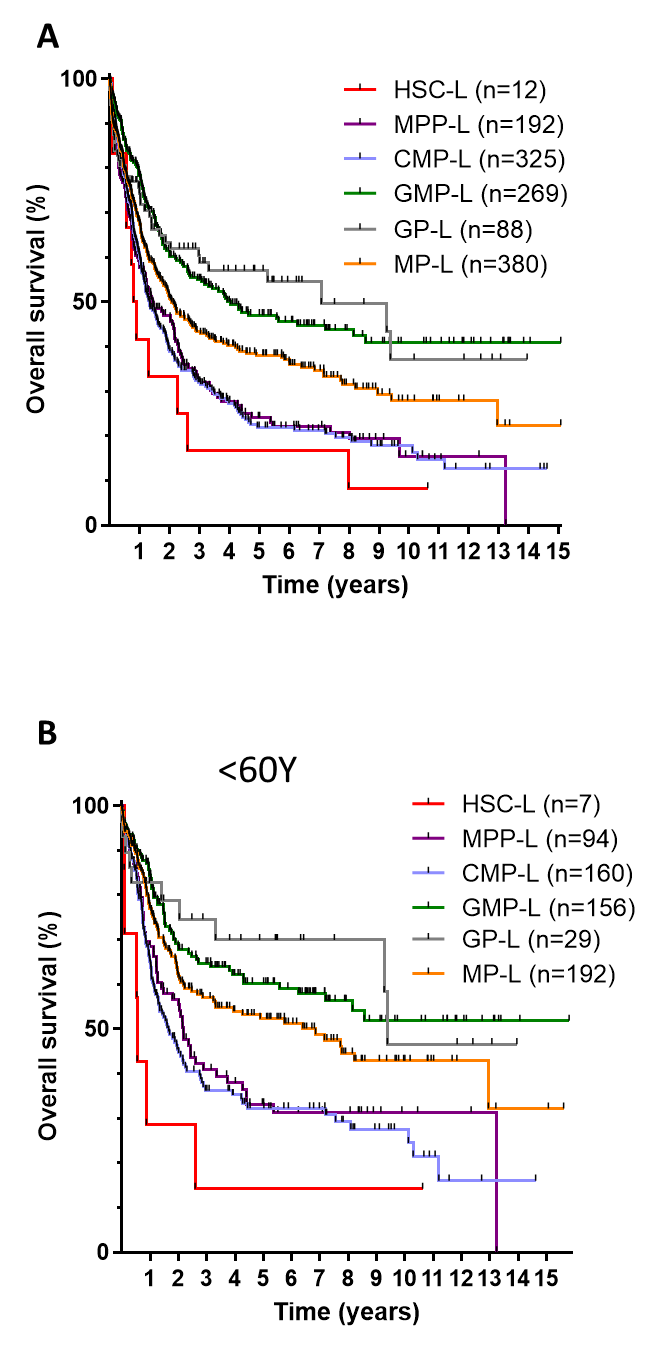


***Supplemental Figure 5: Response to chemotherapy according to the SLA (related to Figure 6).*** (A) Prognostic impact of SLA on overall survival for patients from TUH cohort treated with intensive chemotherapy (n=1266). (B) Prognostic impact of SLA on overall survival for younger patients (<60 years) from TUH cohort treated with intensive chemotherapy (n=638).


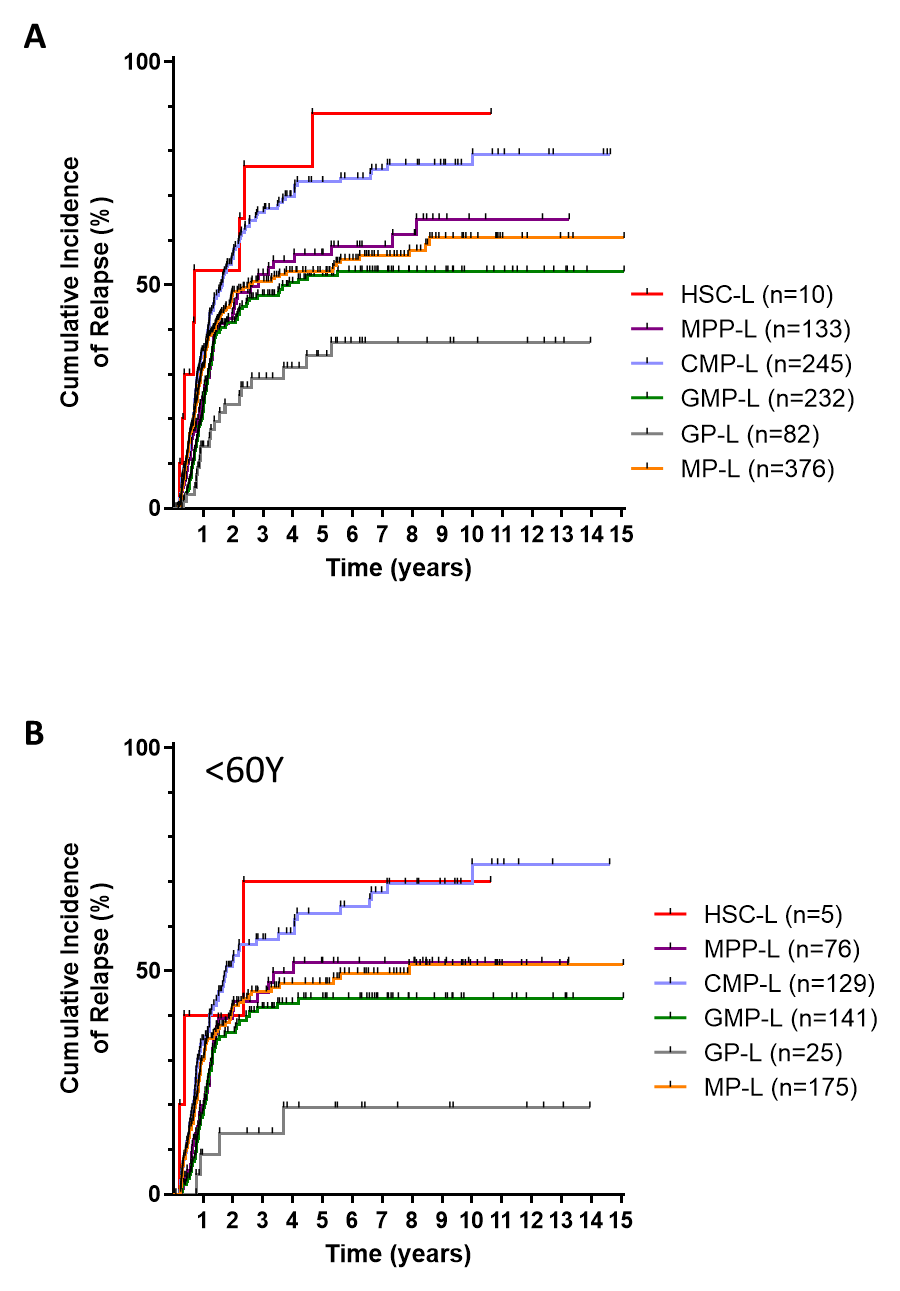


***Supplemental Figure 6: Response to chemotherapy according to the SLA (related to Figure 6).*** (A) Curves of cumulative incidence of relapse in remission in patients treated by intensive chemotherapy (n=1078) according to their SLA. See Table S4 for multivariate analysis results. (B) Curves of cumulative incidence of relapse in remission in younger patients (<60 years) treated by intensive chemotherapy (n=551) according to their SLA.


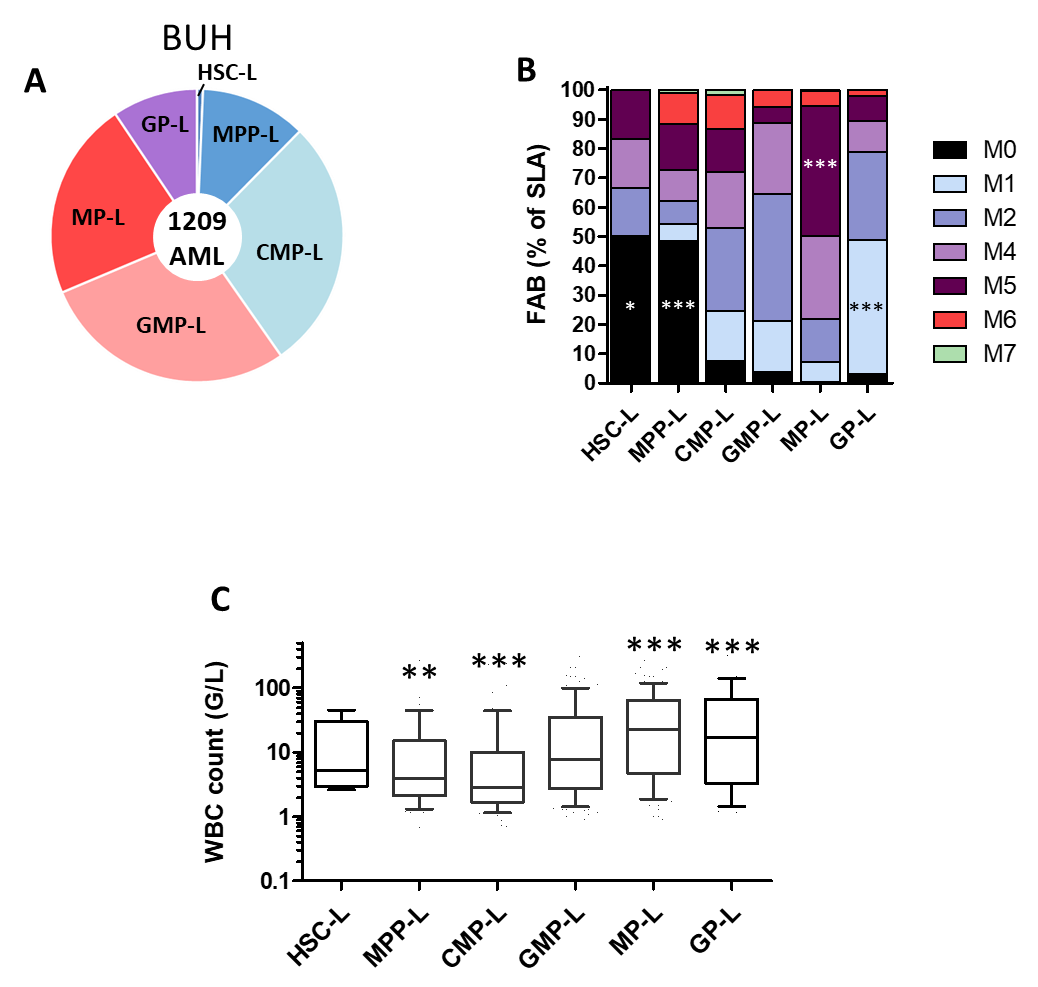


***Supplemental Figure 7: Clinical characteristics of SLA in BUH cohorts***. (A) Pie chart of 1209 AML from BUH cohort segregated according to their SLA. (B) FAB classification according to SLA in BUH cohort. (C) Boxplots of leukocytosis at diagnosis in BUH cohort.


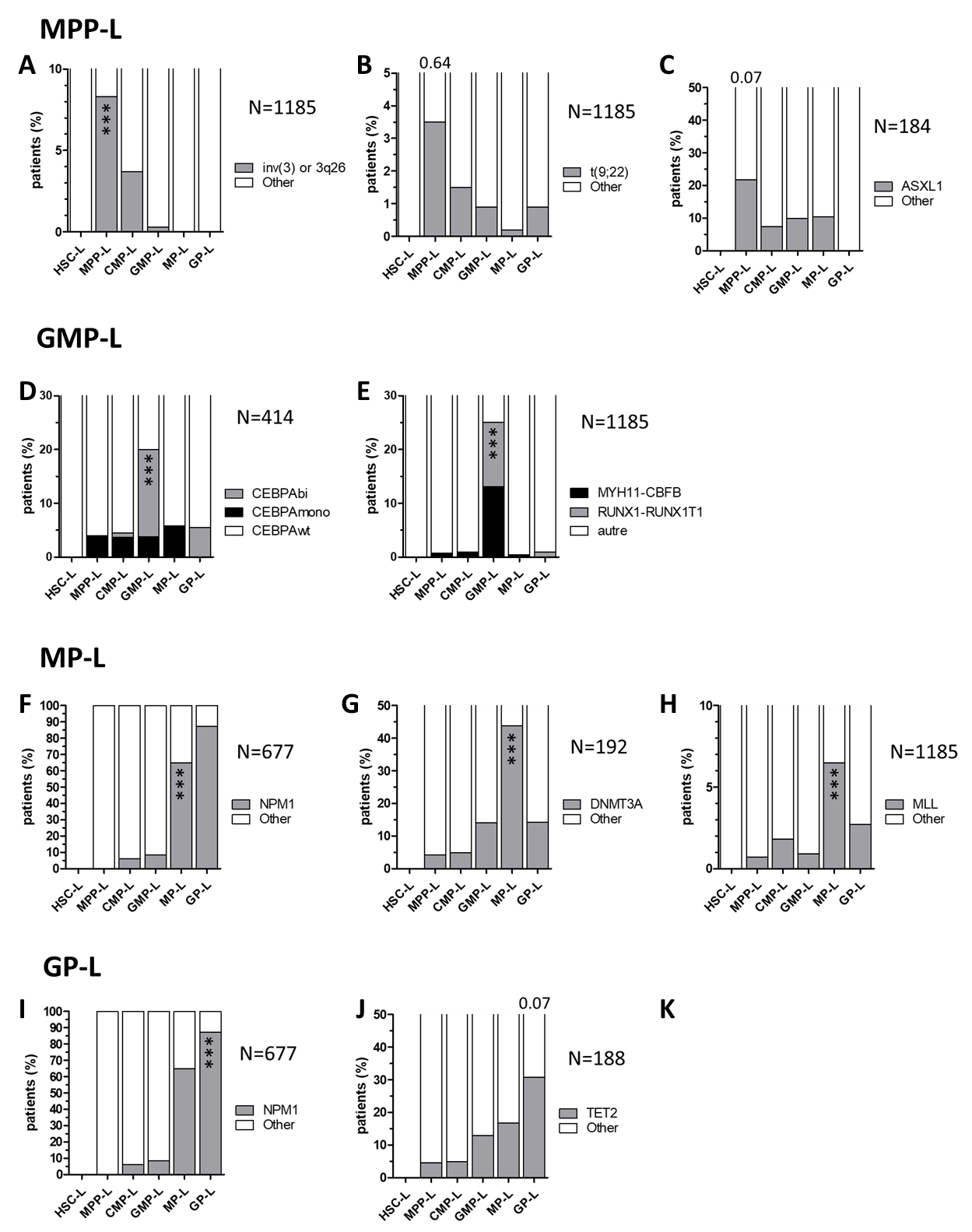


***Supplemental Figure 8: Validation of SLA specific mutations and genetic anomalies in BUH cohort.*** (A-C) Validation of specific mutations and genetic anomalies identified in MPP-L. (A) Percentage of inv(3) or 3q26 anomalies according to SLA. (B) Percentage of t(9;22) anomalies according to SLA. (C) Percentage *ASXL1* mutation according to SLA. (D-E) Validation of specific mutations and genetic anomalies identified in GMP-L. (D) Percentage of *CEBPA* mutations according to SLA. (E) Percentage of CBF anomalies according to SLA. (F-H) Validation of specific mutations and genetic anomalies identified in MP-L. (F) Percentage of *NPM1* mutations according to SLA. (G) Percentage of *DNMT3A* mutation according to SLA. (H) Percentage t(11q23;x) anomalies according to SLA. (I-K) Validation of specific mutations and genetic anomalies identified in GP-L. (I) Percentage of *NPM1* mutation according to SLA. (J) Percentage of *TET2* mutation according to SLA. (K) Percentage *IDH* mutations according to SLA.


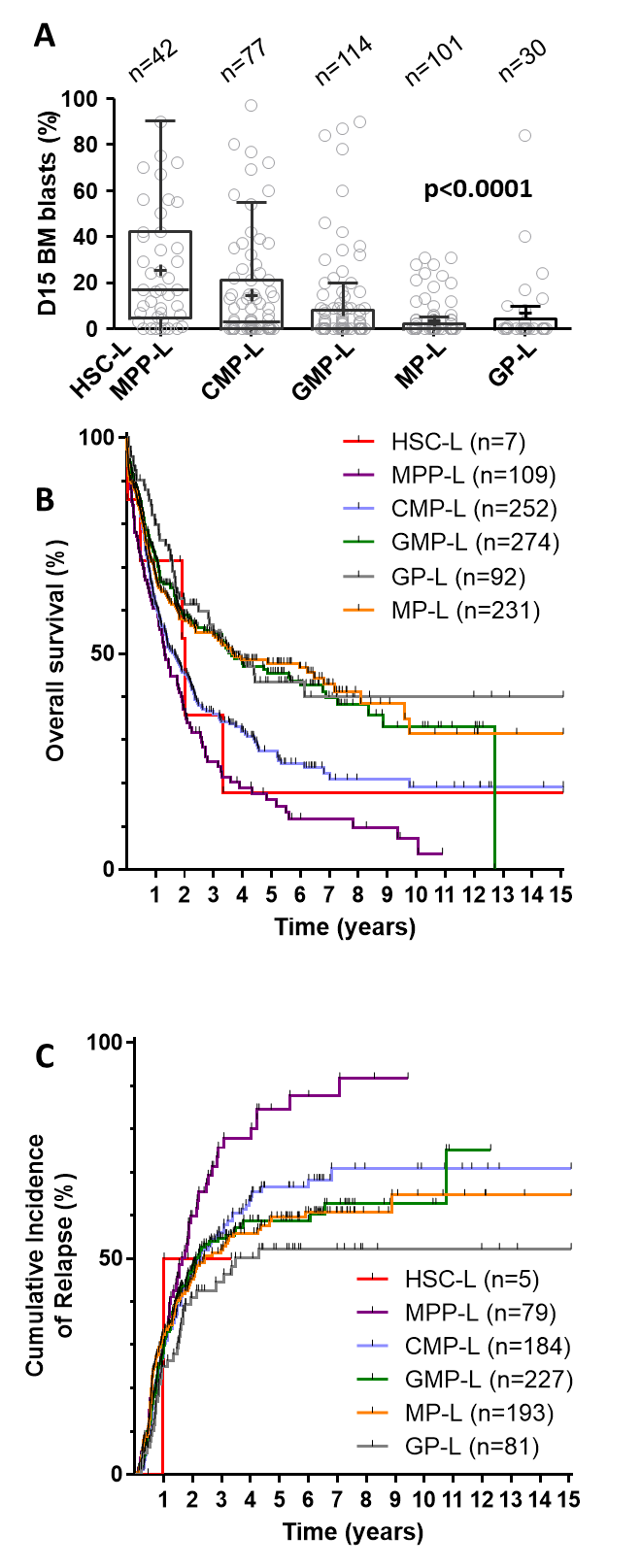


***Supplemental Figure 9: Response to chemotherapy according to the SLA in BUH cohort.*** (A) Early chemosensitivity according to SLA evaluated in patients by measuring the percentage of residual blasts in bone marrow at day 15 of induction chemotherapy (n=364). (B) Prognostic impact of SLA on overall survival for patients from BUH cohort treated with intensive chemotherapy (n=965). See Table S8 for multivariate analysis results. (C) Curves of cumulative incidence of relapse in remission in patients treated by intensive chemotherapy (n=769) according to their SLA.


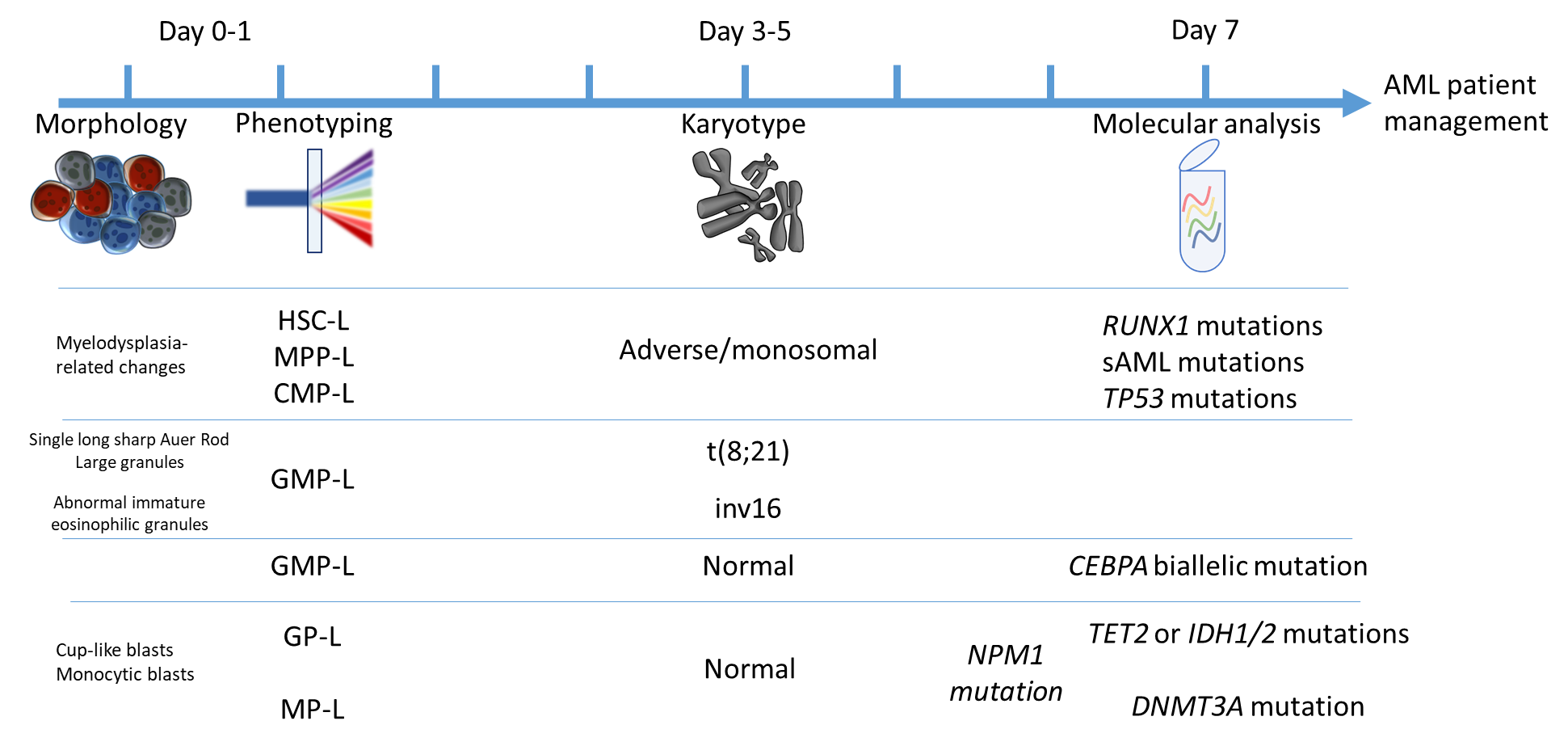


***Supplemental Figure 10: SLA classification in the initial workup of AML patients.*** The SLA classification can be obtained in less than 24 hours and allows, in association with morphological parameters, to anticipate karyotype and molecular biology analyses to improve patient management.

**Supplemental Tables**

**Table S1.** Characteristics of patients from the BUH cohort.

**Table S2**. Early deaths assessment in SLA.

**Table S3.** Landscape analysis of allografted and non-allografted AML (<65 years) in CR according to SLA.

**Table S4.** Comparison of SLA at diagnosis and at relapse

**Table S5.** Cox regression model for overall survival of TUH cohort (related to Figure 6).

**Table S6.** Competing risk regression model for cumulative incidence of relapse of TUH cohort (related to Figure 6).

**Table S7.** Summary of AML patient characteristics according to the SLA classification.

**Table S8.** Cox regression model for overall survival of BUH cohort (related to Supplemental Figure 7).
